# Frequency and significance of post-intubation hypotension during emergency airway management

**DOI:** 10.1186/cc9574

**Published:** 2011-03-11

**Authors:** A Heffner, D Swords, J Kline, A Jones

**Affiliations:** 1 Carolinas Medical Center, Charlotte, NC, USA

## Introduction

Arterial hypotension is known to follow emergency intubation but the significance of this event is poorly described. We aimed to measure the incidence of post-intubation hypotension (PIH) following emergency intubation and determine its association with in-hospital mortality.

## Methods

A retrospective cohort study of endotracheal intubations performed in a large, urban emergency department over a 1-year period. Patients were included if they were >17 years old and had systolic blood pressure (SBP) >90 mmHg for 30 consecutive minutes prior to intubation. Patients were analyzed in two groups: those with PIH defined by SBP <90 mmHg within 60 minutes of intubation, and those with no PIH. The primary outcome was hospital mortality.

## Results

Emergency intubation was performed on 465 patients, of which 336 met inclusion criteria and were analyzed. The median patient age was 49 years, 59% of patients presented with nontraumatic illness and 92% underwent induction with etomidate. PIH occurred in 76/336 (23%) of patients. The median time to first PIH was 11 minutes (IQR 2 to 27). Intubation for acute respiratory failure was the only independent predictor of PIH (OR = 2.1, 95% CI = 1.1 to 4.0). Patients with PIH had significantly higher in-hospital mortality (33% vs. 21%; 95% CI for 12% difference = 1 to 23%) and longer mean ICU length of stay (9.7 vs. 5.9 days, *P *< 0.01) and hospital length of stay (17.0 vs. 11.4 days, *P *< 0.01). Multivariate logistic regression analysis confirmed PIH as an independent predictor of hospital mortality (OR = 1.9, 95% CI = 1.1 to 3.6).

## Conclusions

PIH occurs in nearly one-quarter of normotensive patients undergoing emergency intubation. Intubation for acute respiratory failure is an independent predictor of PIH. PIH is associated with a significantly higher in-hospital mortality and longer ICU and hospital lengths of stay.

